# Is gut microbiota of patients with ALS different from that of healthy individuals?

**DOI:** 10.55730/1300-0144.5825

**Published:** 2024-05-07

**Authors:** Zerin ÖZAYDIN AKSUN, Seyda ERDOĞAN, Ayşe KALKANCI, Elif Ayça ŞAHİN, Tuğba ÇUHADAR, H. Özden ŞENER

**Affiliations:** 1 Department of Neurology, Faculty of Medicine, Ankara University, Ankara, Turkiye; 2 Department of Microbiology, Faculty of Medicine, Gazi University, Ankara, Turkiye

**Keywords:** Amyotrophic lateral sclerosis, ALS, gut microbiota, neurodegenerative diseases, pathogenesis of ALS

## Abstract

**Background/aim:**

Amyotrophic lateral sclerosis (ALS) is a fatal neurodegenerative disease. Several studies have shown that alterations of microbiota increase the risk of neurodegenerative disorders. We aimed to reveal whether there is a difference in the gut microbiota of patients with ALS.

**Materials and methods:**

The participants are divided into three groups. Group 1 comprised patients with ALS. Healthy family members living in the same house of the patients formed Group 2. Lastly, sex- and age-matched healthy people were included in Group 3. Fecal samples were collected in 15-mL falcon tubes and stored at −80 °C. Genomic DNA isolation was performed on samples. Bacterial primers selected from the 16S rRNA region for the bacterial genome and ITS1 and ITS4 (internal transcribed spacer) were used for the identification of DNA. Next generation sequence analysis (NGS) and taxonomic analyses were performed at the level of bacterial phylum, class, order, family, genus, and species. Alpha and beta diversity indexes were used. The linear discriminant analysis (LDA) effect size method (LEfSe) was applied to identify a microbial taxon specific to ALS disease.

**Results:**

The relative abundances of the Succinivibrionaceae and Lachnospiraceae families were significantly lower in patients. The dominant families among patients were Streptococcaceae and Ruminococcaceae, while the dominant families among healthy controls were Bacteroidaceae and Succinivibrionaceae. The LEfSe analysis revealed that four families (Atopobiaceae, Actinomycetaceae, Erysipelatoclostridiaceae, Peptococcacceae) differed significantly between the patients and healthy controls (LDA values> 2.5, p < 0.05).

**Conclusion:**

Comparison with family members living in the same house is the strength of this study. We found that there were changes in the microbiota of the patients, consistent with the literature. Studies that analyze the composition of the gut microbiota in the predisease period may be needed to understand whether dysbiosis is caused by the mechanisms inherent in the disease or whether it is dysbiosis that initiates the disease.

## 1. Introduction

Amyotrophic lateral sclerosis (ALS) is a fatal neurodegenerative disease that affects the upper and the lower motor neurons. The pathogenesis is unclear. It can be sporadic or familial. In sporadic cases, various predisposing genes and environmental factors are implicated for the disease [1]. Imbalance in the gut microbiota composition may be one of the environmental factors. The gut microbiota has an important role in the immune system since it is the main site of exposure to pathological and intrinsic antigens and toxins [2]. The results of some studies have shown that alterations of gut microbiota increase the risk and severity of some neurodegenerative disorders. For instance, the patients with Parkinson’s disease showed a lower abundance of Prevotellaceae members [3] and some microbial groups may be different in patients with Alzheimer’s disease compared to controls [4]. To date, the results of the studies on the relationship between intestinal microbiota and ALS are contradictory [5]. Some researchers documented a lower Firmicutes/Bacteroidetes (F/B) ratio and differential abundances of certain genera in the microbiota composition of patients with ALS compared to controls [6–8], and others suggested that there is no significant taxonomic and metagenomic differences [9, 10]. These conflicting results may be explained in several ways. Primarily, ALS disease shows heterogeneity in terms of genetics, phenotype, and pathophysiological basis. In addition, there are many confounding factors including diet and drugs that may affect the results of those studies.

In this study, we aimed to reveal whether there is a difference by examining the gut microbiota of drug-naïve patients with ALS, their healthy family members with similar nutritional habits, and age- and sex-matched healthy controls.

## 2. Materials and methods

### 2.1. Participants

The participants of this study are divided into three groups. Group 1 included the patients between the ages of 18 and 80 years who were admitted to Ankara University Faculty of Medicine, Neurology Department and diagnosed with definite ALS according to the revised El Escorial criteria. Patients with ALS were not treated with riluzole or the other FDA-approved drug for ALS until fecal samples were collected. Riluzole is an FDA approved drug used in the treatment of ALS, primarily metabolized in the gastrointestinal system. All patients were examined by two neurologists and evaluated with the Revised Amyotrophic Lateral Sclerosis Functional Rating Scale (ALSFRS-R). Healthy family members living in the same house as the patients in Group 1 formed Group 2 as ALS controls, while sex- and age-matched healthy people (not related to patients) from the community were randomly included in Group 3.

There were no individuals who adhered to a vegan or vegetarian diet in any of the groups.

Participants with a history of diarrhea or antimicrobial drug use in the past 3 weeks and with a history of inflammatory bowel disease or history of bowel operations were excluded.

### 2.2. Sample collection

Fecal samples were collected in 15-mL falcon tubes and stored at −80 °C. No information except the patient’s name and surname was written on the sample tube. The laboratory worker was blind to all groups to which the samples belong.

### 2.3. Analysis of samples

Genomic DNA isolation was performed on samples from patients with ALS, their family members, and controls. A special DNA isolation system (The QIAamp DNA Stool Mini Kit) prepared for this purpose was used for fecal samples. Bacterial primers selected from the 16S rRNA region for the bacterial genome and ITS1 and ITS4 panfungal primers selected from the 5.8S rRNA region for the fungal genome were used for the identification of DNA. For this purpose, bacteria-fungus combined Swift Amplicon 16S+ITS Panel (Swift Biosciences) was used. Next-generation sequence analysis (NGS) was applied using the MiSeq platform (Illumina). The results were evaluated with QIIME 1.9.1 software package and Python 3, and compared with SILVA v138 16S-rRNA and Unit v8.2 data. Taxonomic analyses of the groups were performed at the level of bacterial phylum, class, order, family, genus, and species.

### 2.4. Statistical analysis

In this study, three different alpha diversity indexes (Shannon, Evennes, and Faith Phylogenetic Diversity Index) were used to examine the microbial species diversity in each sample. Species diversity among samples was compared with beta diversity analysis. For beta diversity assessment in each group, four different statistical analyses (Bray-Curtis, Jaccard, Weighted UniFrac and Unweighted UniFrac) were performed. The linear discriminant analysis (LDA) effect size (LEfSe) method was applied to identify a microbial taxon specific to ALS disease [11].

## 3. Results

Six patients with ALS (5 males, 1 female) and six healthy family members living in the same house as the patients (4 females, 2 males) were included in this study. Additionally, eight healthy people (5 males, 3 females) who were not related to patients constituted the third group. The mean age was similar in all groups; 58 ± 11.64 in Group 1, 56.2 ± 11.77 in Group 2, and 52.5 ± 7.44 in Group 3. The mean weight was 70.6 kg (SD: 15.96) with BMI of 25.30 (SD: 4.70) in Group 1, 87.8 kg (SD: 22.73) with a BMI of 34.67 (SD: 11.67) in Group 2, and 80.1 kg (SD: 13.7) with a BMI of 27.41 (SD: 2.99) in Group 3. The mean duration of symptoms in patients was 15 months (SD:12.4). The demographic and clinical characteristics of participants are summarized in Tables 1 and 2.

Three patients died due to disease progression during the three-year follow-up period.

Fungal operational taxonomic units (OTUs) (2425381 OTUs) could not be classified into taxon data and were not identified; therefore, we present only the bacterial microbiota results. A total of 1088972 bacterial OTUs were identified.

**Figure 1:**
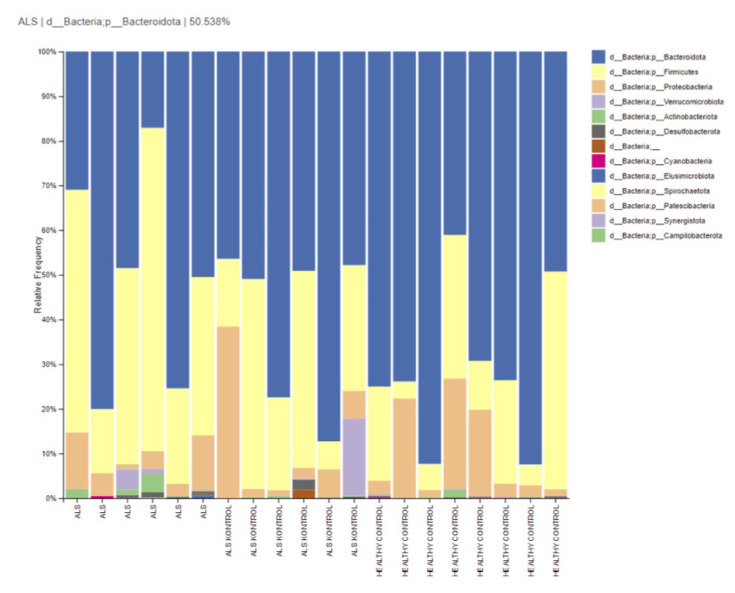
Relative abundances of phylum-level taxa in groups.

Fecal bacterial flora of ALS patients consisted of the phyla Bacteroidetes (48.8%), Firmicutes (42%), and Proteobacteria (6%). The fecal bacterial flora of ALS controls, who were the patients’ relatives, consisted of the phyla Bacteroidetes (59.6%), Firmicutes (26.5%), and Proteobacteria (1%). Healthy controls (HCs) presented Bacteroidetes (69.9%), Firmicutes (18.7%), and Proteobacteria (10.6%). The relative abundance of phylum-level taxonomy was similar (p > 0.05) (Figure 1).

**Figure 2:**
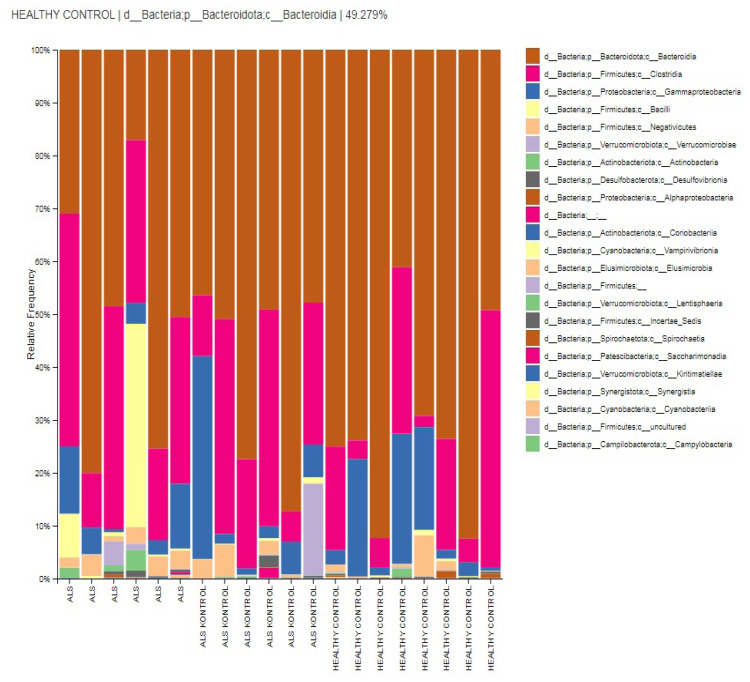
Relative abundances of class-level taxa in groups.

Dominant classes are Bacteroidia (59.9%), Clostridia (24.1%), Bacilli (9.6%), Gammaproteobacteria (6%), and Negativicutes (2.8%) in ALS group. Patients’ relatives presented class Bacteroidia (49.2%), Clostridia (29.7%), Gammaproteobacteria (10.3%), Negativicutes (2.1%), and Bacilli (0.3%). Similarly, HC subjects presented in their gut flora dominantly Bacteroidia (70.3%), Clostridia (17%), Gammaproteobacteria (10.3%), Negativicutes (1.5%), and Bacilli (0.2%). The dominancy of relative abundance of Bacilli in the ALS group is statistically significant (p < 0.05), and according to biomarker analysis, the class Coriobacteriia is considered a biomarker class for patients with ALS (LDA score >4, p < 0.05) (Figures 2 and 6).

**Figure 3:**
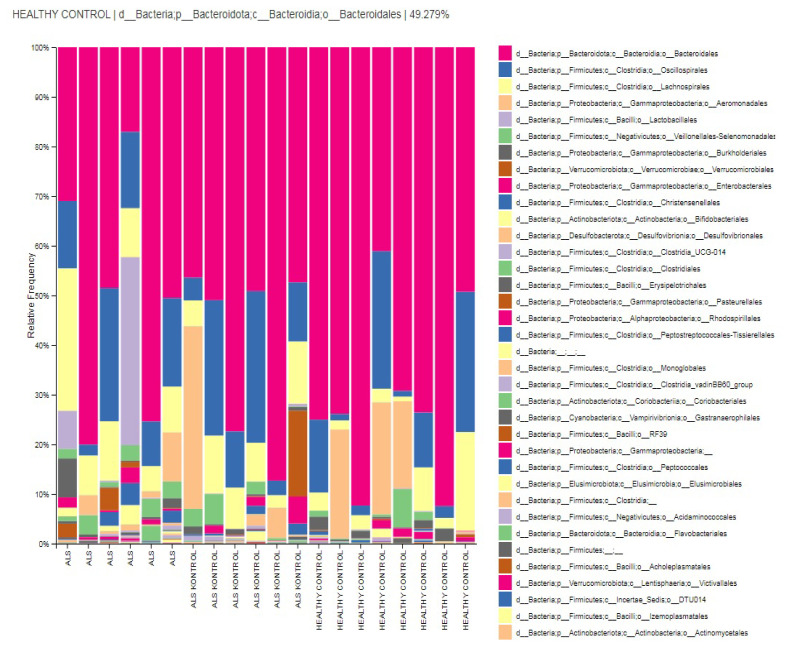
Relative abundances of order-level taxa in groups.

The relative abundances of order-level taxa were Bacteroidales, Oscillospirales, Aeromonadales, Lachnospirales, and Verrucomicrobiales in both ALS patients’ relatives (ALS controls) and HCs group with different percentages. However, taxon Aeromonadales is absent in patients with ALS. Dominant orders were Bacteroidales (52.2%), Oscillospirales (15.2%), Lachnospirales (12.9%), Lactobacillales (9.7%), and Veillonellales-Selenomonadales (3%) in the ALS group. According to the biomarker analysis, the taxon Actinomycetales and taxon Coriobacteriales are considered biomarker orders for patients with ALS (LDA score > 4, p < 0.05) (Figures 3 and 6).

**Figure 4:**
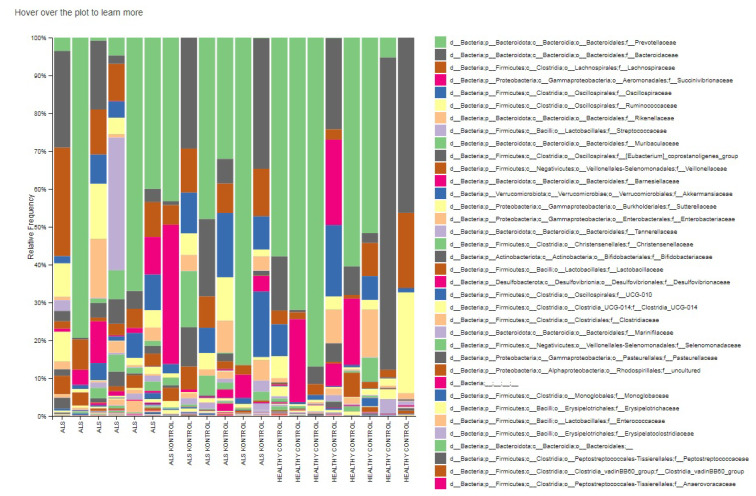
Relative abundances of family-level taxa in groups.

The relative abundances of family level taxa were Prevotellaceae (32.8%), Streptococcaceae (8.1%), Ruminococcaceae (6.1%), Oscillospiraceae (5.4%), Rikenellaceae (3.6%) in the ALS group. Prevotellaceae, Bacteroidaceae, Succinivibrionaceae, Lachnospiraceae, and Oscillospiraceae families were dominant in both patients’ relatives and HCs groups. Succinivibrionaceae and Lachnospiraceae families were absent or in low abundance level in the gut flora of the ALS patients. According to LEfSe biomarker analysis, the taxon Atopobiacea, Actinomycetaceae and the taxon Erysipelatoclostridiaceae were considered biomarker families for patients with ALS (LDA score > 4, p < 0.05) (Figures 4 and 6).

**Figure 5:**
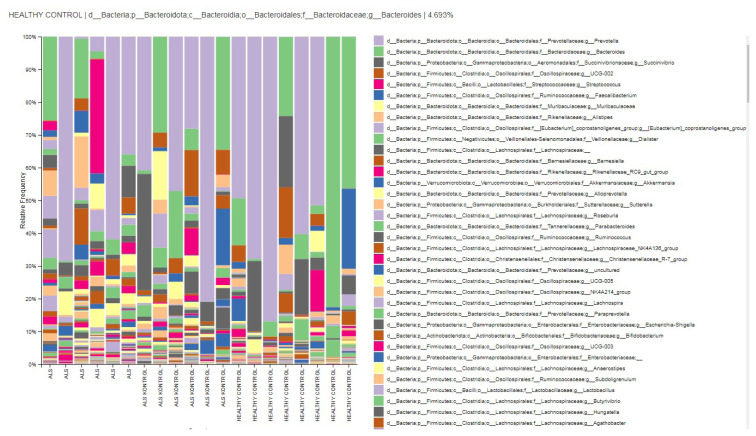
Relative abundances of genus-level taxa in groups.

**Figure 6:**
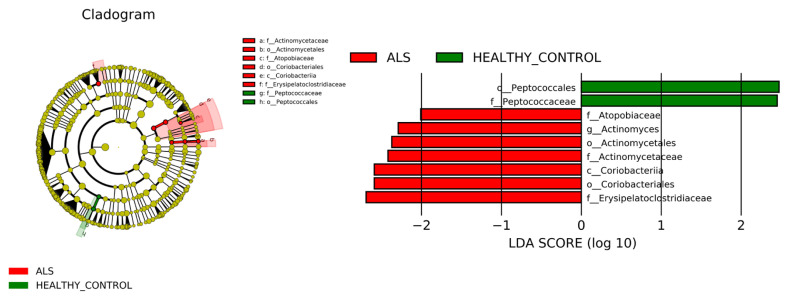
LDA score and cladogram. The cladogram results of LEfSe analysis show taxonomic clades that are differential in abundance, where different circles from the inside to the outside represent different classification levels from domain to species and the larger size of the nodes reflects higher relative abundance. The biomarkers are colored in red and green depending on the group. The bar chart shows the biomarkers with differential abundance between the groups and larger than the preset value (LDA score > 4, p < 0.05). The LDA score indicates the extent to which the corresponding group is affected by the differential microbes.

**Figure 7:**
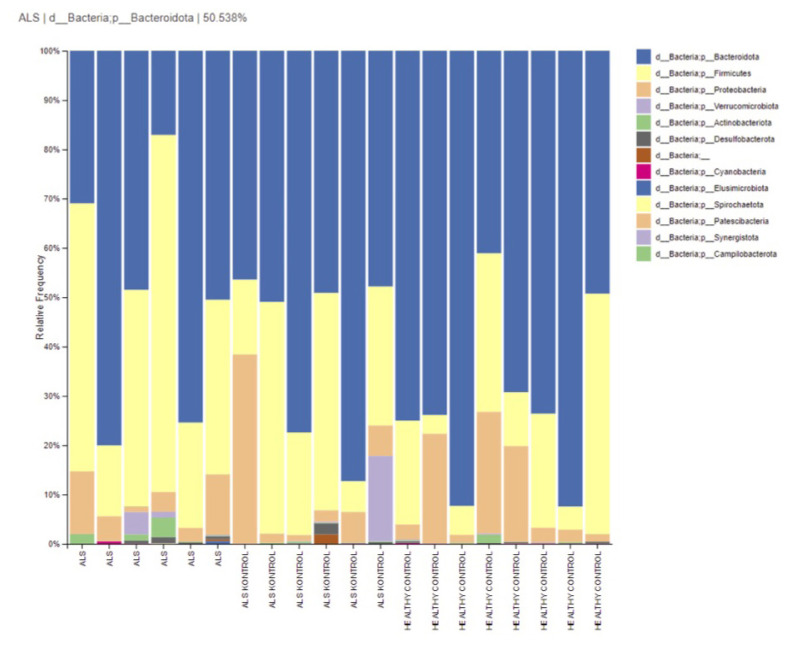
Firmicutes and Bacteroidetes levels of the participants.

The relative abundances of the genus-level taxa were Prevotella (66.9%), Bacteroides (11.9%), Streptococcus (10.1%), Alistipes (3.7%), and Dialister (3.4%) in the ALS group. Prevotella, Bacteroides, Succinivibrio, and Faecalibacterium families were dominat in patients’ relatives (ALS controls) and HCs. Succinivibrio and Faecalibacterium were absent in the gut flora of the ALS patients. According to the LEfSe biomarker analysis, the taxon Actinomyces was considered a biomarker genus for patients (LDA score > 4, p < 0.05) (Figures 5 and 7).

## 4. Discussion

The pathogenesis of neurodegenerative diseases involves a lot of mechanisms such as neuroinflammation, protein aggregates, excitatory overstimulation, mitochondrial dysfunction, toxin exposure, and impaired axonal transport [12]. One of the most discussed issues recently has been dysbiosis. Dysbiosis is defined as an imbalance in microbiota properties or functions because of decreased microbial diversity, increased pathogenic strains, and loss of beneficial strains [13–15]. It is hypothesized that the increased intestinal permeability in ALS in the interaction between microbiota and central nervous system facilitates the passage of toxins from the intestinal lumen into the blood, leading to an increase in circulating lipopolysaccharides and an immune response [16]. In other words, an unhealthy gut may create a neuro-toxic environment by disrupting the neurovascular structure and causing leakage in the blood-brain barrier and increasing the risk of developing neurodegenerative diseases such as ALS [5, 14]. The view that these responses are elicited by changes in the relative abundance of some bacteria is gaining in importance. An increase in the genus Dorea, increase in Anaerostipes, decrease in the genus Prevotella, decrease in the family Lachnospiraceae, decrease in yeast abundance, and increase in *E. coli* and Enterobacteria are among those implicated in the pathogenesis [13, 17].

We performed this study to determine if there was a disease-specific change by including family members with the same nutritional characteristics. We did not find any significant difference in relative abundances at phylum level between groups. Bacteroidetes and Firmicutes are the two most abundant phyla in the adult gut, with difference in relative abundance of one affecting the other [14].

There are conflicting results regarding the Firmicutes/Bacteroidetes (F/B) ratio in studies conducted in patients with ALS. While there are opinions that suggest there is an increase [15, 16], or decrease [7, 8], there are also studies suggesting that there is no change [9, 10]. In our study, F/B ratios were found to be 0.9, 0.44, 0.27 in Groups 1, 2, and 3, respectively. Although the rates were different from each other, they were not statistically significant. It was determined that there was a decrease in the F/B ratio in 4 of our patients, an increase in 2, a decrease in 4 of the patient’s relatives, and a decrease in 7 of the healthy controls. A higher F/B ratio was associated with an increased risk of death [10]. Although the number of participants is limited, the F/B ratio of 2 of our 3 patients who died is low. F/B ratio was low in 11 of 14 patients in the healthy group. Additionally, F/B changes were also detected in healthy participants. In this case, our results are contradictory in terms of F/B ratio.

In our study, the relative abundances of Succinivibrionaceae and Lachnospiraceae families were significantly lower in samples taken from patients. Lachnospiraceae and its beneficial products (e.g., short-chain fatty acids) maintain the integrity of the intestinal epithelial barrier, thus making it a family of beneficial bacteria [13]. It has been shown that there is a significant decrease in the relative abundance of the family Lachnospiraceae in patients [7]. In parallel with the opinion that there is a difference in the clustering of bacterial profile between ALS and healthy controls [17], the dominant families in the intestinal microbiota of patients in our study were Streptococcaceae and Ruminococcaceae, while the dominant families of the healthy controls were Bacteroidaceae and Succinivibrionaceae. Presence of Ruminococcus has been interpreted as a risk factor for the disease [6]. In our patients, the Ruminococcaceae family was dominant.

LEfSe analysis showed that four families (Atopobiaceae, Actinomycetaceae, Erysipelatoclostridiaceae, Peptococcacceae) differed significantly between the patients and healthy subjects (LDA values > 2.5, p < 0.05). The dominant bacterial families in Group 1 were Erysipelotrichaceae, Atopobiaceae, and Actinomycetaceae. The bacterial family Erysipelatoclostridiaceae has been reported to be very immunogenic [18]. An increased abundance of the family Actinomycetaceae has been found in the oral microbiota of Alzheimer’s patients [19].

To understand whether dysbiosis occurs by the mechanisms inherent in the disease or dysbiosis initiates the disease, studies conducted in the predisease period may be needed.

In a study conducted for this purpose, gut dysbiosis was identified long before the onset of motor dysfunction and immune cell activation in mice models [20]. Blacher and colleagues found a different microbiome composition in presymptomatic period in transgenic SOD1 mice [21].

In this study, which included patients and their healthy relatives, we investigated whether there was a clear difference between family members with similar nutritional habits. The small number of our patients is a limitation, but it can be attributed to the conditions new diagnosis and not using FDA-approved drugs in ALS, which may serve as reasons for this limitation. The detailed nutritional characteristics of the participants were not questioned, which can be considered another limitation.

## Figures and Tables

**Table 1:** Patients’ characteristics.

Group 1 (Patients)	Sex	Age	Height (cm)	Weight (kg)	BMI (kg/m^2^)	Symptom duration (months)	ALSFRS-R	Disease onset site
1	M	67	166	67	24.3	6	23	Bulbar
2	M	43	174	74	24.4	6	24	Limb
3	M	62	168	60	21.3	6	22	Limb
4	F	62	150	54	24.0	12	23	Limb
5	M	70	173	69	23.05	36	22	Bulbar
6	M	44	170	100	34.6	24	42	Limb
Mean (SD)		58 (11.64)	166.8 (8.8)	70.6 (15.96)	25.3 (4.7)	15 (12.4)	26 (7.9)	

**Table 2:** Controls’ characteristics.

Number		Sex	Age	Height (cm)	Weight (kg)	BMI (kg/m^2)^
Group 2 (patients’relatives, ALS controls)	1	F	-	-	-	-
	2	F	41	160	69	26.95
	3	F	60	160	70	27.3
	4	M	64	173	75	25.06
	5	F	69	160	110	42.97
	6	M	47	150	115	51.1
Mean (SD)			56.2 (11.77)	160.6 (8.17)	87.8 (22.73)	34.67 (11.67)
Group 3 (healthy controls)	1	M	44	177	105	33.5
	2	F	51	150	58	25.78
	3	M	48	164	81	30.1
	4	F	55	165	70	25.7
	5	F	60	164	75	27.89
	6	M	42	188	88	24.9
	7	M	62	178	80	25.2
	8	M	58	179	84	26.2
Mean (SD)			52.5 (7.44)	170.6 (12.02)	80.1 (13.7)	27.41 (2.99)
